# MAGE genes: Prognostic indicators in AL amyloidosis patients

**DOI:** 10.1111/jcmm.14475

**Published:** 2019-06-20

**Authors:** Yang Liu, Lei Wen, Ling Ma, Ying Kang, Kai‐Yan Liu, Xiao‐Jun Huang, Guo‐Rui Ruan, Jin Lu

**Affiliations:** ^1^ Peking University People's Hospital, Peking University Institute of Hematology Beijing China; ^2^ Beijing Key Laboratory of Hematopoietic Stem Cell Transplantation Beijing China; ^3^ Collaborative Innovation Center of Hematology, Peking University Beijing China; ^4^ Peking‐Tsinghua Center for Life Sciences Academy for Advanced Interdisciplinary Studies, Peking University Beijing China; ^5^ Collaborative Innovation Center of Hematology Soochow University Suzhou China

**Keywords:** amyloid light‐chain amyloidosis, cancer‐testis antigen gene, real‐time quantitative polymerase chain reaction

## Abstract

A high frequency of MAGE‐CT (cancer testis) antigens are expressed in Multiple Myeloma (MM) patients; however, in other plasma cell dyscrasias, their potential function remains unclear. We measured the expression of MAGE‐CT genes (MAGE‐C1/CT7, MAGE‐A3, MAGE‐C2/CT10) in 105 newly diagnosed amyloid light‐chain (AL) amyloidosis patients between June 2013 and January 2018 at Peking University People's Hospital using real‐time quantitative polymerase chain reaction. In the newly diagnosed AL patients, the positive expression rates of patients with MAGE‐C1/CT7, MAGE‐C2/CT10 and MAGE‐A3 were 83.8% (88/105), 56.71% (38/67) and 22.0% (13/59) respectively. There was no significant correlation between organ propensity and MAGE‐CT gene expression. Changes in the MAGE‐C1/CT7 levels were consistent with a therapeutic effect. The expression levels of MAGE‐C1/CT7, MAGE‐C2/CT10 and MAGE‐A3 provide potentially effective clinical indicators for auxiliary diagnoses and monitoring treatment efficacy in AL amyloidosis patients.

## 1 INTRODUCTION

Amyloidosis is a disorder that is characterized by misfolded insoluble protein fibrils deposited in a variety of tissues and organs. Amyloid light‐chain (AL) systemic amyloidosis is one form of systemic amyloidosis and is caused by plasma cell dyscrasia. Current therapies, including novel drugs and autologous stem cell rescue, significantly improve the survival of these patients. However, some patients are still not responsive to anti‐plasma cell therapy.^1^, ^2^, ^3^ Therefore, it is necessary to understand the biological characteristics of tumour plasma cells in AL amyloidosis. The mechanisms responsible for tumorigenic plasma cells in AL amyloidosis involve a series of genetic alterations in the bone marrow microenvironment that promote tumour growth and the failure of the immune system to recognize it.^4^, ^5^

The cancer‐testis families of tumour‐associated antigens (CT antigens) were originally discovered in patients with malignant melanoma. These antigens are expressed in a broad range of human tumours, while they are limited to developing germ cells in normal tissue and occasionally the placenta.^6^ Additionally, MAGE‐CT antigens are able to elicit cytotoxic T cells and humoural responses. Because CT antigens show restricted normal tissue expression and are highly immunogenic, they are attractive targets for diagnostic value as tumour markers and immunotherapeutic approaches in cancer patients. Regarding Multiple Myeloma (MM), our previous study and other research show that MAGE‐CT antigens are expressed with a frequency of 59%‐92.3% in symptomatic MM patients.^7^, ^8^, ^9^ However, the study of CT antigens is mainly concentrated in myeloma, and there is little research in areas of other plasma cell dyscrasia including AL amyloidosis.^10^

To investigate the relationship between MAGE‐CT antigen expression and AL amyloidosis, we measured the messenger ribonucleic acid expression of primary bone marrow specimens from newly diagnosed AL amyloidosis patients by real‐time polymerase chain reaction (RT‐PCR) and analysed their relationship with the prognosis of patients.

## 2 METHODS

### 2.1 Patients

Between June 2013 and Jan 2018, 105 AL amyloidosis patients who underwent MAGE CT gene detection were enrolled in this study. AL amyloidosis was diagnosed by the presence of Congo Red‐positive fibril deposition upon biopsy and evidence of monoclonal protein upon serum protein electrophoresis, serum/urine immunofixation electrophoresis, or free light chain (FLC) analysis. Immunofluorescence or immunoelectron microscopy was used to identify the AL subtype. Organ involvement was assessed according to the consensus criteria.^11^ Baseline data at diagnosis were extracted from medical records, whereas follow‐up information was recorded after each visit. CD138‐enriched (fluorescence in situ hybridization, FISH) panels for cytogenetics from bone marrow were collected from patients. FISH panels included t(4;14), t(14;16), t(11;14), del17p, +1q21. If the proportion of bone marrow clonal plasma cells ≥10% in one patient without bone destruction, hypercalcinaemia and tubular nephropathy AL amyloidosis was still diagnosed. The definition of plasma cell dyscrasia was established according to the definitions of the international myeloma working group.^12^, ^13^, ^14^ Informed consent was obtained from all patients prior to their enrolment in the study. The study design adhered to the principles of the Helsinki Declaration and was approved by the ethics committee of Peking University People's Hospital.

### 2.2 Real‐time quantitative PCR for MAGE‐CT antigens

Bone marrow samples were collected from patients with AL amyloidosis during routine diagnostic procedures at the Peking University People's Hospital. The real‐time quantitative PCR technique in our laboratory was described in detail previously.^7^ The 10‐μL PCR mixture contained 5 μL 1× TaqMan^®^ Universal PCR Master Mix (Applied Biosystems, Foster City, California), 400 nmol/L primers, 250 nmol/L fluorescent probes and 150‐500 ng cDNA. PCR was performed with the ABI PRISM^®^ 7500 FAST Sequence Detection System (Applied Biosystems) at 50°C for 2 minutes and 95°C for 10 minutes, followed by 50 cycles at 95°C for 15 seconds and 60°C for 1 minute. The expression levels of the three CT antigen genes (MAGE‐C1/CT7, MAGE‐C2/CT10, MAGE‐A3) were quantified by qPCR using the abelson (*ABL*) genes as internal controls. The primers and probes were designed using Primer Express 2.0 software (Applied Biosystems, California) as follows: ABL (forward 5'‐CCGCTGACCATCAATAAGGAA‐3', reverse 5'‐GATGTAGTTGCTTGGGACCCA‐3' and probe 5'‐FAM‐CCATTTTTGGTTTGGGCTTCACACCATT‐TAMARA‐3'); MAGE‐C1/CT7 (forward 5'‐TTGTCTTCTGGGAACCTTGACTC‐3', reverse 5'‐TGAGGGACACATACATCCTAAAAGC‐3' and probe 5'‐FAM‐ACTGCCTGGGCCTCCTCTGCTGT‐BHQ‐3'); MAGE‐C2/CT10 (forward5'‐GTGTGAGGCACACAGCCTAAAG‐3', reverse 5'‐GGAGGCATGACGACTTCTTCA‐3' and probe 5'‐FAMAGGAGTCAAGGCCTGTTGGATCTCATCA‐BHQ‐3'); and MAGE‐A3 (forward 5'‐GGTGAGGAGGCAAGGTTCTGA‐3', reverse, 5'‐GTGCTGACTCCTCTGCTCAAGAG‐3' and probe, 5'‐FAM‐AGATCTGCCAGTGGGTCTCCATTGCC‐BHQ‐3'). All CT antigens were quantified against the ABL standard curve in bone marrow specimens to decrease experimental error. The detection sensitivity was approximately 1‐10 copies in the plasmid DNA standards and 10^−4^‐10^−5 ^copies in bone marrow specimens.

### 2.3 Treatment response and outcome

A total of 66 out of 105 patients received bortezomib‐based chemotherapy. Haematological response and organ response were evaluated according to the consensus guidelines.^15^

### 2.4 Statistical analysis

The *χ* ^2^ or Fisher's exact tests were used for categorical variables, whereas a *t* test or nonparametric test was used for continuous variables. Standard deviation for the positive distribution and quartiles for the non‐normal distribution were calculated to compute the standard. End‐points were calculated at the time of last contact; the overall survival (OS) was defined from the first time of relapse to the last contact or the time of death. A survival curve was generated using the Kaplan‐Meier method. Comparison of survival was performed by the log‐rank test. A *P* < 0.05 denoted statistical significance. All *P* values were two‐sided. All statistical analyses were performed with SPSS 23.0 (Inc, Chicago, IL).

## 3 RESULTS

### 3.1 Baseline characteristics of patients

The median age of all the patients at baseline was 60.8 years (range, 37‐85) with a male/female ratio of 2.18 (Table 1). The frequency of organ involvement was kidney (79.2%), heart (68.3%), liver (13.9%), peripheral nerve (11.4%) and intestine (9.5%). Of the patients, 68.3% had more than one organ involved. The median value of NT‐proBNP was 3885.4 pg/mL (range, 5‐35000); the median value of troponin I (cTnI) was 0.129 µg/L (range, 0.001‐1.474). A total of 28.9% of patients were Mayo 2004 cardiac stage III, 22.1% of patients were Mayo 2012 stage III, and 16.9% of patients were Mayo 2012 stage IV patients at diagnosis. We also included 128 patients with active myeloma, 14 with plasma cell leukaemia, nine with smouldering myeloma, 67 with monoclonal gammopathy of undetermined significance (MGUS) and 17 with active myeloma complicated with AL amyloidosis.

**Table 1:** Baseline characteristics in AL amyloidosis

Baseline characteristics in AL	Values
Sex (Male, %)	72 (68.6%)
Age (median, range, y)	60.8 (37‐85)
Subtype of light chain (kappa/lambda)	29/76
Organ involvement (%)	
Kidney/Heart/Liver/Intestinal/Nerve	79.2%/68.3%/13.9%/11.4%/9.5%
>1 organ involved by AL (%)	68.3%
NT‐proBNP	
Median (range, pg/mL)	3885.4 (5‐35000)
≥8500 pg/mL (%)	9.5%
cTnI	
Median (range, ug/l)	0.129 (0.001‐1.474)
≥0.07 μg/L (%)	35.2%
IVS (mm)	1.1 (0.6‐2.1)
Hb (median, range, g/L)	123 (66‐169)
Creatinine(median, range, μmol/L)	124.4 (22‐491)
ALP (median, range, U/L)	117.4 (22‐1035)
LDH (median, range, U/L)	226.5 (109‐522)
β2MG(median, range, μg/mL)	4.5 (1.6‐9.0)
BMPC (median, range, %)	8.7% (0%‐40%)
Mayo Stage (2004)	
I/II/III (%)	25.6%/45.6%/28.9%
Mayo Stage (2012)	
I/II/III/IV	35.1%/26.0%/22.1%/16.9%

Abbreviations: ALP, alkaline phosphatase; β2MG, β2microglobulin; BMPC, bone marrow plasma cell; cTnI, cardiac troponin; Hb, haemoglobin; IVS, interventricular septum; LDH, lactate dehydrogenase; NT‐proBNP, N‐terminal fragment of the pro‐brain natriuretic peptide.

### 3.2 Expression frequency and organ tropism of three CT antigen genes in AL amyloidosis

As shown in Table 2, in the newly diagnosed AL patients, 83.8% (88/105) of specimens expressed MAGE C1/CT7. The rank of the positive expression rate in the newly diagnosed AL was MAGE‐C1/CT7 (83.8%, 88/105) > MAGE‐C2/CT10 (56.71%, 38/67) > MAGE‐A3 (22.0%, 13/59). For MAGE‐C1/CT7, the median value was 7.28% (range: 0.005%‐82.27%). If we divided the expression intensity of MAGE into four groups (<0.1%, 0.1%‐1%, 1%‐10%, 10%‐100%), there were 28 (31.8%), 16 (18.2%), 30 (34.1%) and 14 (15.9%) patients in the above groups respectively. There were 14 AL amyloidosis patients with dFLC <50 mg/dL, among whom 12 patients (85.7%) had positive expression for MAGE‐C1/CT7. The average level of MAGE‐C1/CT7 expression was 2.587% (range: 0.01%‐22.16%).

**Table 2:** Correlation of the clinic‐pathological characteristics of patients with AL amyloidosis with the expression of CT antigens

	MAGE‐C1/CT7 (n = 105)			MAGE‐C2/CT10 (n = 67)			MAGE‐A3 (n = 59)		
	+	−	*P*	+	−	*P*	+	−	*P*
PC value	9.102 (4‐11.375)	5.406 (3‐8.75)	0.107	10.962 (5‐15)	6.089 (4‐9)	0.008	12.594 (6.5‐16.75)	7.847 (4‐9.5)	0.011
LDH	228.273 (179.75‐271.25)	200.688 (174.75‐217.75)	0.088	223.385 (168‐234)	232.893 (181.5‐296.5)	0.542	220.188 (152‐250.5)	231.245 (183‐276)	0.250
β2MG	4.294 (2.773‐5.848)	4.798 (2.75‐8)	0.631	4.204 (2.37‐5.81)	4.942 (3.3‐8)	0.173	4.077 (2.31‐6.49)	4.674 (2.78‐6.81)	0.398
dFLC	431.54 (58.38‐466.41)	550.18 (67.92‐396)	0.988	505.39 (84.68‐571.5)	569.28 (69.15‐769.5)	0.936	325.91 (136.49‐421.08)	596.71 (61.11‐961.90)	0.660
NT‐pro BNP	4112.88 (386.6‐4879.5)	2657.05 (107‐2671)	0.278	4296.0 (180.5‐4865.8)	4056.23 (280.6‐4726.0)	0.374	3552.16 (54.4‐4907.0)	5939.8 (250.1‐3563.0)	0.593
cTnI	0.1361 (0.0085‐0.1533)	0.081 (0.001‐0.0325)	0.038	0.085 (0.003‐0.108)	0.164 (0.003‐0.151)	0.695	0.122 (0.001‐0.193)	0.115 (0.004‐0.084)	0.974
t(11;14)	14.3% (10/70)	23.1% (3/13)	0.423	14.3% (5/35)	333% (8/24)	0.083	7.1% (1/14)	25.6% (11/43)	0.142
Del(17p)	1.4% (1/74)	0 (0/15)	0.651	0	0	—	0	0	—
1q21	25.7% (19/74)	13.3% (2/15)	0.305	29.7% (11/37)	23.1% (6/2)	0.558	66.7% (10/15)	15.2% (7/46)	<0.001

Abbreviations: β2MG, β2microglobulin; cTnI, cardiac troponin; dFLC, difference between involved and uninvolved free light chain; LDH, lactate dehydrogenase; NT‐proBNP, N‐terminal fragment of the pro‐brain natriuretic peptide; PC, plasma cell.

There was no significant correlation between organ propensity and MAGE‐CT gene expression. Additionally, in each targeted organ subgroup, the rank of the positive expression rate in the newly diagnosed AL was MAGE‐C1/CT7 > MAGE‐C2/CT10 > MAGE A3, which was the same as in the overall cohort.

### 3.3 Spectrum of MAGEC1/CT7 expression in different plasma cell dyscrasias

**Figure 1:**
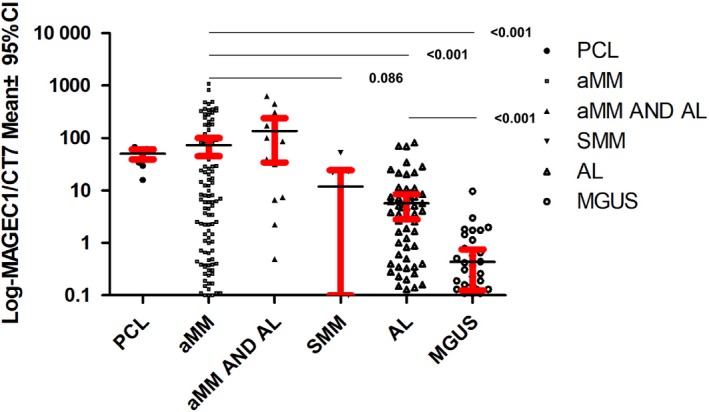
The spectrum of MAGE‐C1/CT7 expression in different plasma cell dyscrasia. The level of MAGE‐C1/CT7 gene expression in plasma cell leukaemia (PCL), aMM, aMM complicated with AL, SMM, AL and monoclonal gammopathy of undetermined significance (MGUS) was, 50.20%, 78.50%, 152.5%, 15.38%, 6.90% and 0.64% respectively

The expression of the MAGE‐C1/CT7 gene was significantly highest in the plasma cell leukaemia and active myeloma groups and lowest in the MGUS group (Figure 1). The level of MAGE‐C1/CT7 gene expression in MM patients was higher than in the amyloidosis and MGUS groups (*P* < 0.001). Additionally, the level of MAGE‐C1/CT7 gene expression in the amyloidosis group was higher than in the MGUS group (*P* < 0.001).

### 3.4 Correlation between CT antigens and prognostic factors in AL amyloidosis

The percentages of BM plasma cell infiltration positively correlated with the expression levels of MAGE‐C2/CT10 (*P* = 0.008) and MAGE‐A3 (*P* = 0.011). We did not find a correlation between MAGE‐CT antigens and dFLC or NTproBNP. Additionally, there was no correlation between the β2‐microglobulin (β2 MG), Lactate Dehydrogenase (LDH)^16^, ^17^ and MAGE‐CT genes. The level of cTnI correlated with MAGE‐C1/CT7 (*P* = 0.038).

### 3.5 MAGE‐C1/CT7 change in different response groups

**Figure 2:**
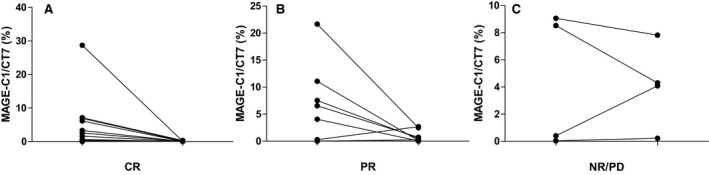
MAGE‐C1/CT7 change in different response groups. MAGE‐C1/CT7 expression levels correlate with the clinical course of AL amyloidosis. (A), The expression levels of MAGE‐C1/CT7 decreased in 15 patients whose clinical efficacy was complete remission (CR). (B), The expression levels of MAGE‐C1/CT7 decreased in 7 out 9 patients whose clinical efficacy was partial remission (PR). (C), The expression levels of MAGE‐C1/CT7 increased or stable in 3 out 4 patients whose clinical efficacy was NR (no remission)/PD (progression disease).

A longitudinal analysis was performed on 28 AL patients during follow‐up. Changes in the disease state from the clinical course of each patient were divided into three groups: Complete Remission (15 paired samples); Partial Remission (9 paired samples); Stable Disease/Progression Disease (PD, 4 paired samples). Most patients showed very good correlation between the changing levels of dFLC (difference between the involved and uninvolved light chain) and MAGE‐C1/CT7 gene expression (Figure 2). Of importance, a clinically significant decrease in serum FLC was associated with a decrease in MAGE‐C1/CT7 expression. The level of dFLC in one patient decreased; however, the MAGE‐C1/CT7 of this patient increased. This female patient was 50 years old with Mayo 2012 stage III. The baseline‐free light lambda was 1592.5 mg/L, whereas the dFLC was 1583.2 mg/L. The baseline MAGE‐C1/CT7 was 0.28%. In 20 September 2016, we used CyborD (bortezomib, cyclophosphamide, dexamethasone) for 2 cycles, after which the decrease in amylogenic FLC were 1137 mg/dL; however, the level of MAGEC1/CT7 increased to 2.67%. The patient received the same CyborD for another two cycles, after which changes in amylogenic FLC (from the best) increased by 117.6%. The patient was determined to be PD and died on 01 April 2017.

### 3.6 Relationship between MAGE antigen and OS

**Figure 3:**
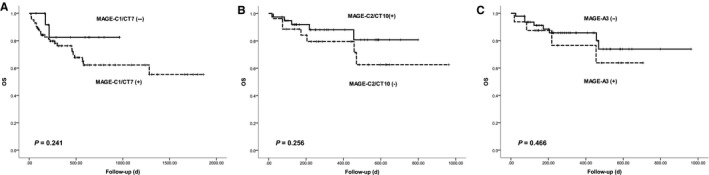
Overall Survival according to the expression of MAGE‐CT antigens. (A), MAGE‐C1/CT7; (B) MAGE‐C2/CT10; (C) MAGE‐A3

After a median follow‐up duration of 1266 days (range, 18‐1863), the median survival was not reached for either group, and the 1‐ and 2‐year OS were 77.1% and 65.5% respectively. There was no significant difference in overall survival among the patients whose MAGE‐CT antigens were positive or negative (Figure 3).

## 4 DISCUSSION

Our results indicated that CT antigens were potentially effective molecular markers of AL amyloidosis and have clinical implications for monitoring treatment efficacy.

We found that CT antigens (CTA) were commonly expressed in AL amyloidosis. The most prevalent CTA was MAGE‐C1/CT7, which we identified in 88/105 (83.8%) cases. The high frequency of MAGE‐CT expression in AL amyloidosis has clinical significance. Our results indicated that CT antigens were potentially effective molecular markers of AL amyloidosis and have clinical implications for auxiliary diagnoses. The prevalence of MAGE‐CT expression in our AL series was higher than in one previous study of AL amyloidosis, in which 66% positivity for C1/CT7 using immunohistochemistry was observed. The discrepancy was probably due to the different detection methods. Our qPCR protocol provided a reliable and sensitive method for the quantification of MAGE‐C1/CT7, MAGE‐C2/CT10, MAGE‐A3 expression levels.^7^ The detection sensitivity was approximately 1‐10 copies for plasmid DNA standards and 10^−4^‐10^−5^ copies in bone marrow specimens with a high level of sensitivity.^18^

The quantification of MAGE‐C1/CT7 antigens positively correlated with individual IgH levels in MM patients (data not shown). These results suggested that MAGE‐CT antigens were potential markers for the tumour burden of plasma cell dyscrasia including AL amyloidosis. Minimal residual disease (MRD) is trending to become a new surrogate research end‐point, and MAGE‐CT antigens are a new MRD marker that is less time‐consuming to use than traditional RT‐PCR.^19^

The change in MAGE‐CT gene expression paralleled the change in FLC, which was the same as that in myeloma.^7^, ^18^, ^20^ In addition, one patient showed changes in MAGE before FLC changes. These results suggested that MAGE‐C1/CT7 has the potential to detect disease relapse/progression at an earlier stage than the standard clinical monitoring method. These results also showed that the changes in MAGE and FLC could be a good supplement to the monitoring of curative efficacy, not only to monitor the pathogenicity FLC of the plasma cells but also to analyse the changes in the antigen expressed by the plasma cell itself, which may be more comprehensive for the evaluation of some patients.

For AL amyloidosis patients with dFLC <50 mg/L, it was difficult to evaluate efficacy through serological testing during the follow‐up period.^21^, ^22^ In total, 12 patients with dFLC <40 were tested for MAGE simultaneously. The positive frequency was 85.7%, and the average level was 2.59%. It was suggested that MAGE gene detection may be a good alternative monitoring method for this subgroup of patients.

Another contribution of this work was that we reported the difference in the expression intensity of the MAGE gene in different plasma cell dyscrasias. It was found that the expression of MAGE‐C1/CT7 was the strongest in plasma cell leukaemia and weakest in MGUS. Additionally, the expression of MAGE‐C2/CT10 and A3 genes correlated with the plasma cell percentage in AL amyloidosis. All these observations suggested that MAGE‐CT antigens correlated with plasma cell proliferation, which was in accordance with previous reports that MAGE CT antigens were highly expressed in advanced myeloma and less expressed in early stage and MGUS stage.^20^, ^23^ Our results related to amyloidosis and those of previous studies of myeloma suggested a novel association between these antigens and the dysregulation of plasma cell cycling.

It was reported that MAGE‐C1/CT7 and MAGE‐A3 played an important role in promoting the survival of myeloma cells and clonogenic precursors by reducing the rate of apoptosis.^24^ Additionally, MAGE‐C1/CT7 played a role in the regulation of the myeloma cell cycle. Silencing MAGE‐C1/CT7 resulted in a statistically significant increase in the percentage of myeloma cells in G_0_/G_1_ phase and significantly decreased the number of cells in the G_2_/M phase of the cell cycle.^25^ In addition, some studies have shown an association between the expression of CTAs and a phenotype of resistance to chemotherapy treatments.^26^

The prognostic role of the three CT antigens in AL amyloidosis was analysed. In addition to plasma cell (PC) percentage, we found that none of the MAGE CT genes correlated with β2MG, LDH or NT‐proBNP, which are prognostic indictors for AL amyloidosis. The expression level of MAGE‐C1/CT7 correlated with the level of cTnI. Chromosomal abnormalities, including 1q21, t (11; 14), were associated with poor survival rates.^27^ In this study, patients with MAGE A3 expression had more frequent 1q21 amplification. We did not find any correlation between MAGE CT gene expression and a propensity to specific affected organs (organ tropism). Organ tropism has been reported to be partially related to PC clones derived from particular IGLV genes.^28^, ^29^, ^30^, ^31^ Our observations in this study suggested that MAGE genes may not be potential factors regulating organ tropism. Because the overall survival for AL amyloidosis is very complex and mainly relies on the amount of amylogenic‐free light chain and the degree of damage to the heart, we did not find any correlation between MAGE CT gene expression and overall survival. Due to the short follow‐up time and limited cases, these conclusions need to be re‐evaluated in larger sample sizes and during longer follow‐up times.

Vaccine therapy is emerging in MM but not in AL amyloidosis.^23^, ^32^, ^33^, ^34^ Low tumour burden and low tumour cell proliferative index are two advantages for immunotherapy in AL amyloidosis.^35^ The specificity to malignant plasma cells and the common expression of these antigens in AL amyloidosis strongly suggest that they are promising targets for vaccine immunotherapy. Formulating vaccines with MAGE‐CT antigens is an attractive strategy.

In conclusion, our study demonstrated that MAGE‐CT genes, especially MAGE C1/CT7, were commonly expressed in AL amyloidosis, and the expression levels of MAGE CT genes could potentially be used as clinical indicators for auxiliary diagnoses and monitoring treatment efficacy in AL patients. However, these conclusions still need further verification through prospective studies with larger sample sizes. Further investigation into the function of MAGE CT genes in AL amyloidosis is warranted to reveal novel therapeutic targets.

## CONFLICT OF INTEREST

The authors declare that there is no conflict of interest.

## AUTHOR CONTRIBUTIONS

Jin Lu and Guo‐Rui Ruan designed the study. Jin Lu performed data analysis. Guo‐Rui Ruan did the RT‐PCR examination. Yang Liu performed the data collection, and drafted the manuscript. Jin Lu, Xiaojun Huang and Kai‐Yan Liu provided significant input on the manuscript and data analysis. Lei Wen, Ying Kang and Ling Ma helped to collect data and draft the manuscript. All authors read and approved the final manuscript.

## Data Availability

The data that support the findings of this study are available from the corresponding author upon reasonable request.
